# Integrative analysis of efferocytosis- and invasion-related genes as potential biomarkers and therapeutic targets in breast cancer

**DOI:** 10.1007/s12672-025-03346-w

**Published:** 2025-08-05

**Authors:** Jing Yang, Rong Zhang, Lamei Sun, Cong Wang, Ming Feng, Bin Su, Lixin Jiang

**Affiliations:** 1 Department of General Surgery, Jiangyin Hospital Affiliated to Nanjing University of Chinese Medicine, Wuxi, China; 2 Department of Breast Surgery, Affiliated Hospital of Nanjing University of Chinese Medicine, Nanjing, China; 3 Department of Traditional Chinese Medicine, Jiangyin Nanzha Community Health Service Center, Wuxi, China; 4 Department of Oncology, Wuxi Hospital Affiliated to Nanjing University of Chinese Medicine, Wuxi, China

**Keywords:** Breast cancer, Bioinformatics, Efferocytosis, Invasion, Biomarker, Differentially expressed genes

## Abstract

Breast cancer remains a primary source of cancer-related mortality among females worldwide. This investigation sought to evaluate the distinctive expression patterns of genes linked to efferocytosis and invasion in breast cancer and their prognostic implications. Through bioinformatics analyses, a robust prognostic risk modelwas developed. Breast cancer datasets were procured and processed from The Cancer Genome Atlas (TCGA), Gene Expression Omnibus (GEO), and GeneCards databases. Differential expression analysis was executed utilizing DESeq2, identifying genes with|logFC| >1 and p-value < 0.05. Efferocytosis-related genes and invasion-related genes were curated from GeneCards and PubMed, resulting in 127 overlapping genes. A prognostic risk model was developed utilizing univariate Cox regression, Least Absolute Shrinkage and Selection Operator regression, and multivariate Cox regression analyses. A sum of 7860 differentially expressed genes was ascertained in the TCGA-BRCA dataset, comprising 4130 elevated and 11,990 reduced expressions. Among them, 32 efferocytosis- and invasion-related genes exhibited differential expression, including *ANO6* and *PLGRKT*. Univariate Cox regression pinpointed *ANO6* and *PLGRKT* as significant prognostic markers. The Least Absolute Shrinkage and Selection Operator regression yielded the risk score formula: Risk score = *ANO6* × (0.328) + *PLGRKT* × (-0.277). Kaplan-Meier survival analysis suggested a notable difference in survival outcomes between high-risk and low-risk cohorts (p-value < 0.01). Multivariate Cox analysis confirmed risk score, age, NStage subgroups N1-N3, and TStage subgroup T4 as statistically significant prognostic predictors. Functional enrichment analysis suggested that *ANO6* and *PLGRKT* were implicated in biological processes like bleb assembly and the positive regulation of phagocytosis. Gene Set Enrichment Analysis identified notable pathway associations, encompassing the KEAP1/NFE2L2 Pathway and *TP53* Regulation of Metabolic Genes. In conclusion, the developed prognostic risk model effectively predicts survival outcomes in patients with breast cancer, with *ANO6* and *PLGRKT* being pivotal in tumor progression. These observations provide essential knowledge for therapeutic intervention strategies and enhanced clinical care in breast cancer.

**Supplementary Information:**

The online version contains supplementary material available at 10.1007/s12672-025-03346-w.

## Introduction

Breast cancer (BRCA) remains a principal source of cancer-related mortality among females worldwide, recording roughly 2.31 million newly diagnosed instances and 665,684 fatalities in 2022. The global burden is expected to rise further, markedly impacting patients’ quality of life and survival rates [1, 2]. Standard treatments, such as surgery, radiation, chemotherapy, and hormone therapy, have considerably improved survival outcomes [3]. However, these therapies often cause substantial side effects and exhibit limited effectiveness across all patient populations, underscoring the need for more precise and personalized therapeutic approaches.

An increasing body of research highlights the role of efferocytosis- and invasion-related genes in cancer. Efferocytosis refers to the process by which phagocytic cells clear and degrade apoptotic cells, a pathway capable of enabling tumor-associated macrophages (TAMs) to shift toward the M2 phenotype, thereby promoting tumor progression [4]. A study highlights that the efferocytosis-related gene (*IL33*) influences BRCA progression by regulating cytotoxicity and metabolic pathways in CD8 + T cells. *IL33*, identified as a key prognostic marker, suppresses tumor growth and enhances immune-mediated tumor destruction, underscoring the dual role of efferocytosis in both promoting immunosuppression and revealing therapeutic vulnerabilities [5]. Invasion-related genes are integral to cancer cell metastasis, a key factor in cancer-related mortality [5, 6]. Another study reveals that Collagen I triggers a force-dependent Yap activation loop in basal-like breast cancer cells, independent of K14 expression. Yap drives transcriptional programs that enhance Collagen I alignment and tension, creating a feed-forward cycle to sustain collective invasion. Active Yap is critical for leader cell selection and invasion in 3D models and mouse mammary fat pads, highlighting its role as a central mechanotransducer in metastasis [6]. Dysregulation of these genes has been linked to poor prognosis in diverse cancers, encompassing colorectal, lung, and ovarian cancers [7–10]. However, their specific roles and clinical implications in BRCA remain poorly defined, necessitating further investigation.

This study employed a bioinformatics approach to examine the differential expression of efferocytosis- and invasion-related genes in BRCA and their prognostic relevance. TCGA, GEO, and GeneCards datasets were employed to ascertain differentially expressed genes (DEGs) between BRCA and control specimens. A prognostic risk model (PRM) was established utilizing Cox and LASSO regression analyses, and its effectiveness was confirmed through Receiver Operating Characteristic (ROC) curves and Kaplan-Meier (KM) survival analysis. Functional enrichment analyses (FEAs) were implemented to uncover the molecular processes linked to these genes, and protein-protein interaction (PPI) networks were constructed to examine their interrelationships.

Our results demonstrated that the identified genes are critical in BRCA advancement and clinical outcomes. The PRM exhibited strong precision in forecasting patient outcomes, indicating its potential clinical utility. FEA suggested that these genes are implicated in key biological processes (BPs) and pathways, including cell cycle regulation, apoptosis, and immune response, all essential for cancer development and progression [11–13]. The PPI network analysis further emphasized the complex interactions among these genes, offering potential insights into novel therapeutic interventions.

## Methodologies and materials

### Data collection

The BRCA information was procured through The Cancer Genome Atlas (TCGA) portal (https://portal.gdc.cancer.gov/) [14] utilizing the TCGAbiolinks R package (Version 2.30.0) [15]. Following removing cases with incomplete clinical records, the analysis incorporated 1062 BRCA specimens and 110 non-tumor specimens. The information underwent standardization to FPKM (Fragments Per Kilobase per Million) format, while associated clinical records were extracted from the UCSC Xena platform (https://xena.ucsc.edu/) [16]. Table 1 provides detailed information.

**Table 1:** Baseline table with BRCA patients characteristics

Characteristics	Overall
*MStage, n (%)*	
M0	892 (84%)
M1&MX	170 (16%)
*NStage, n (%)*	
N0	513 (48.3%)
N1	357 (33.6%)
N2	116 (10.9%)
N3	76 (7.2%)
*TStage, n (%)*	
T1	268 (25.2%)
T2	623 (58.7%)
T3	138 (13%)
T4	33 (3.1%)

Additionally, the BRCA datasets GSE42568 [17] and GSE29431 were downloaded from the GEO database (https://www.ncbi.nlm.nih.gov/geo/) [18] utilizing the R package GEOquery (Version 2.70.0) [19]. The analyzed specimens within these datasets originated from Homo sapiens breast tissue. Comprehensive dataset characteristics are depicted in Table 2. The GSE42568 dataset, based on the GPL570 platform, included 104 BRCA samples and 17 control samples, while the GSE29431 dataset, also utilizing the GPL570 platform, contained 54 BRCA specimens and 12 control specimens. All specimens were incorporated into the analysis.

**Table 2:** GEO microarray chip information

	GSE42568	GSE29431
Platform	GPL570	GPL570
Species	Homo sapiens	Homo sapiens
Tissue	Breast	Breast
Samples in BRCA group	104	54
Samples in control group	17	12
Reference	PMID: 23,740,839	\

The R package sva initially addressed batch effects within BRCA datasets GSE42568 and GSE29431, yielding merged GEO datasets. To verify batch effect elimination efficacy, distribution boxplots illustrated expression values prior to and following batch effect adjustment. Furthermore, PCA plots demonstrated low-dimensional feature distribution before and after batch effect elimination. The distribution boxplot and PCA visualizations confirmed successful batch effect removal from the BRCA datasets. Detailed information is depicted in Figure S1.

The GeneCards database (https://www.genecards.org/) [20], an extensive resource for human gene information, was employed to ascertain efferocytosis- and invasion-related genes (EIRGs). Using the keywords “Efferocytosis” and “Invasion,” 55 efferocytosis-related genes (ERGs) were initially obtained by filtering for “Protein Coding” genes with a relevance score > 2. Similarly, 2233 invasion-related genes (IRGs) were identified using the same criteria. Additionally, a search for “Efferocytosis” in PubMed (https://pubmed.ncbi.nlm.nih.gov/) [21–23] resulted in 324 ERGs, while searching for “Invasion” yielded 1,029 IRGs [24–26]. After merging and removing duplicates, 326 ERGs and 3,042 IRGs were obtained. The overlap between these collections produced 127 EIRGs. Detailed information is depicted in Table S1.

The sva R package (Version 3.50.0) [27] was utilized to eliminate batch variations across the GSE42568 and GSE29431 datasets, generating the Merged GEO datasets. This consolidated information encompassed 158 BRCA specimens and 29 normal specimens. The unified GEO information underwent normalization through the limma R package (Version 3.58.1) [28], with probe annotations being standardized. To assess the batch correction efficiency, principal component analysis (PCA) [29] was executed on expression matrices before and after batch adjustment. PCA functions as a dimension reduction approach that captures essential characteristics from multidimensional information, converting it to lower dimensions and displaying these elements in 2D or 3D representations for verification.

### BRCA-associated efferocytosis- and invasion-associated DEGs

Specimens from the TCGA-BRCA database were split between BRCA and reference populations. Gene expression variance examination was performed utilizing DESeq2 R software [30] (Version 1.42.0) to evaluate both cohorts. Differentially expressed genes were selected using|logFC| >1 criteria and p-value < 0.05. Genes exhibiting logFC > 1 and p-value < 0.05 were designated elevated DEGs, whereas those showing logFC < −1 and p-value < 0.05 were classified as reduced DEGs. The analytical outcomes were depicted in a volcano visualization created with ggplot2 R software (Version 3.4.4).

To identify efferocytosis- and invasion-related DEGs (EIRDEGs), the DEGs from the TCGA-BRCA dataset (|logFC| >1, p-value < 0.05) were intersected with EIRGs. A Venn diagram was constructed to identify the overlap, and the resulting EIRDEGs were illustrated via a heatmap developed by the R package R package (Version 1.0.12).

### Development of BRCA PRM

To construct the PRM in TCGA, univariate and multivariate Cox regression analyses were executed employing the R package survival (Version 3.5-7) [31], incorporating clinical information to evaluate the prognostic impact of EIRDEGs. To determine whether these EIRDEGs were independent prognostic factors, univariate Cox regression analysis was first executed. Variables with a p-value < 0.1 were subsequently incorporated into the multivariate Cox regression analysis.

LASSO regression analysis was subsequently executed utilizing the glmnet R package [32] (Version 4.1-8) with the parameter family = “cox” based on the EIRDEGs identified in the univariate Cox regression analysis. Ten iterations were implemented in the process. This methodology facilitated PRM gene selection. LASSO regression, which extends beyond traditional linear regression, incorporates a penalization component (lambda × absolute value of slope) to minimize model overadaptation and strengthen its broad applicability. The analytical outcomes were depicted through both the PRM illustration and the variable progression graph. The ultimate risk calculation utilized this equation, derived from LASSO regression coefficients:

$$\:\text{r}\text{i}\text{s}\text{k}Score\:=\:\sum\:_{i}Coefficient\:\left({gene}_{i}\right)\text{*}mRNA\:Expression\:\left({gene}_{i}\right)$$

### Prognostic analysis of BRCA PRM

To evaluate the PRM’s effectiveness, determine optimal models, eliminate inferior alternatives, and establish ideal thresholds within individual models, a time-dependent Receiver Operating Characteristic (ROC) curve [33] was implemented. The survivalROC R package (Version 1.0.3.1) facilitated the creation of time-dependent ROC curves and Area Under the Curve (AUC) calculations, utilizing risk scores and overall survival (OS) data from the TCGA-BRCA dataset’s BRCA specimens. This evaluation focused on predicting survival outcomes at 3-, 4-, and 5-year intervals. AUC values spanned from 0.5 to 1, with higher values signifying enhanced diagnostic capability. Values exceeding 0.5 suggested that molecular expression facilitated event occurrence; ranges of 0.5–0.7 indicated minimal accuracy, 0.7–0.9 suggested moderate accuracy, while values above 0.9 demonstrated high accuracy. The survival R package [31] (Version 3.5-7) was employed for KM curve [34] analysis to examine OS differences between high and low-risk cohorts, with the KM curve generated from risk score data.

The outcomes of univariate and multivariate Cox regression examinations were depicted through a forest plot, demonstrating risk score expression and clinical parameter patterns in both analyses. A Nomogram [35], which portrays the correlations among various independent variables through separate line segments, was established utilizing the rms R package (Version 6.7-1), derived from multivariate Cox regression outcomes, emphasizing the connections between risk scores and clinical parameters.

A Calibration Curve illustrated the framework’s predictive precision by contrasting actual outcomes against model-projected probabilities across various scenarios. The calibration examination evaluated the PRM’s precision and differentiation capabilities using multivariate Cox risk calculations. Furthermore, Decision Curve Analysis (DCA) [36], a practical approach for assessing clinical prediction frameworks, diagnostic evaluations, and molecular indicators, was implemented through the ggDCA R package (Version 1.1) to generate DCA visualizations, providing additional validation of the BRCA PRM’s precision and discriminatory power.

### Gene ontology (GO) and pathway (KEGG) enrichment analysis

GO analysis [37] represents a prevalent method for extensive functional enrichment investigations, incorporating BP, Cellular Component (CC), and Molecular Function (MF). The KEGG [38] functions as a leading repository, offering extensive data on genomes, biological pathways, diseases, and drugs. For GO and KEGG enrichment analysis, we used *ANO6* and *PLGRKT* as input gene sets. These two genes are differentially expressed genes (EIRDEGs) associated with efferocytosis and invasion identified by LASSO regression. To ensure the accuracy and biological relevance of the enrichment analysis results, we selected a screened gene set as the background genes. The background gene set was obtained from the R package “org.Hs.eg.db”, which provides relatively comprehensive gene function annotation information. GO and KEGG pathway enrichment evaluations were conducted on the screened Model Genes utilizing the clusterProfiler R package [39] (Version 4.10.0), implementing a p-value < 0.05 selection standard.

### Gene set enrichment analysis (GSEA)

The BRCA dataset (TCGA-BRCA) underwent stratification into high-risk and low-risk cohorts utilizing median risk scores. Gene expression variance analysis utilized DESeq2 (Version 1.42.0), with findings displayed through volcano plots generated via ggplot2 R package (Version 3.4.4), emphasizing genes meeting|logFC| >2 and p-value < 0.05 criteria. The pheatmap R package (Version 1.0.12) generated heat maps displaying expression levels of genes satisfying|logFC| >2 and p-value < 0.05 parameters. GSEA examination encompassed all genes within BRCA specimens utilizing clusterProfiler R package (Version 4.10.0) [39]. GSEA configurations incorporated a 2020 seed, establishing gene set parameters between 10 and 500 genes. Gene sets were acquired from the Molecular Signatures Database (MSigDB), specifically utilizing c2.cp.all.v2022.1.hs.symbols.gmt data. GSEA selection utilized a p-value < 0.05 threshold.

### Gene set variation analysis (GSVA)

GSVA [40] functions as an unsupervised, distribution-free technique that quantifies gene set enrichment across specimens by transforming gene expression data into sample-specific pathway scores. This methodology determines whether particular pathways exhibit differential enrichment among various specimens. Gene sets were obtained from MSigDB [41], specifically utilizing the c2.cp.v2023.2.hs.symbols.gmt file. GSVA implementation utilized the GSVA R package (Version 1.50.0) on the complete gene collection from the TCGA-BRCA dataset’s BRCA specimens, emphasizing pathway enrichment distinctions between elevated and reduced risk categories. GSVA selection criteria employed a p-value threshold of 0.05, implementing Benjamini-Hochberg (BH) adjustment for p-value correction.

### PPI network

The PPI network encompasses interacting proteins that execute essential roles in BPs, encompassing signal transduction, genetic expression control, metabolic processes, and cell cycle modulation. A comprehensive examination of protein interactions within biological systems is essential for comprehending protein roles, biological signaling mechanisms, and metabolic pathways under various physiological states, encompassing pathological conditions. This investigation also helps illuminate protein functional associations. The GeneMANIA database [42] (https://genemania.org/) generates functional gene hypotheses, examines gene collections, and ranks genes for subsequent functional investigation. When given query genes, GeneMANIA discovers functionally comparable genes by analyzing extensive genomics and proteomics information, assigning weights to datasets based on query gene predictive value. Furthermore, GeneMANIA forecasts gene functions by identifying genes likely to share functional characteristics with query genes utilizing their interactions. The PPI network was established by identifying functionally similar genes to the Model Genes through the GeneMANIA platform.

### Differential expression verification and ROC curve analysis

BRCA samples from the TCGA-BRCA dataset were split into high-risk and low-risk cohorts utilizing the median value of the risk score procured from the prognosis model. To examine the distinctions in Model Gene expression among these cohorts, a comparative visualization was generated utilizing the Model Gene’s expression levels. The diagnostic effectiveness of Model Gene expression was assessed through ROC curve visualization and AUC calculation utilizing the R package pROC [43] (Version 1.18.5). The AUC metric typically extends from 0.5 to 1, with elevated values denoting superior diagnostic capability. An AUC surpassing 0.5 suggests that molecular expression might facilitate event occurrence, with values of 0.5–0.7 denoting minimal accuracy, 0.7–0.9 suggesting intermediate accuracy, and above 0.9 representing superior accuracy.

Risk scores for BRCA specimens from consolidated GEO datasets were ascertained through risk coefficient calculations. BRCA specimens were categorized into high-risk and low-risk cohorts utilizing median risk score values. To examine Model Gene expression variations between these categories, a comparative expression visualization was developed. The diagnostic utility of Model Gene expression underwent evaluation through ROC curve visualization and AUC calculation utilizing the R package pROC (Version 1.18.5).

### Statistical analysis

R software (Version 4.3.0) facilitated data manipulation and examination. When evaluating continuous variables across two cohorts, the independent Student’s t-test determined statistical significance for normally distributed data, except where noted otherwise. Non-normally distributed variables underwent Mann-Whitney U test (Wilcoxon Rank Sum test) analysis. The Kruskal-Wallis test examined differences among three or more cohorts. Correlation coefficients between molecular variables were established through Spearman correlation analysis. Statistical evaluations employed two-sided testing unless specified differently, with significance established at *p* < 0.05.

## Results

### Technology roadmap

**Fig. 1:**
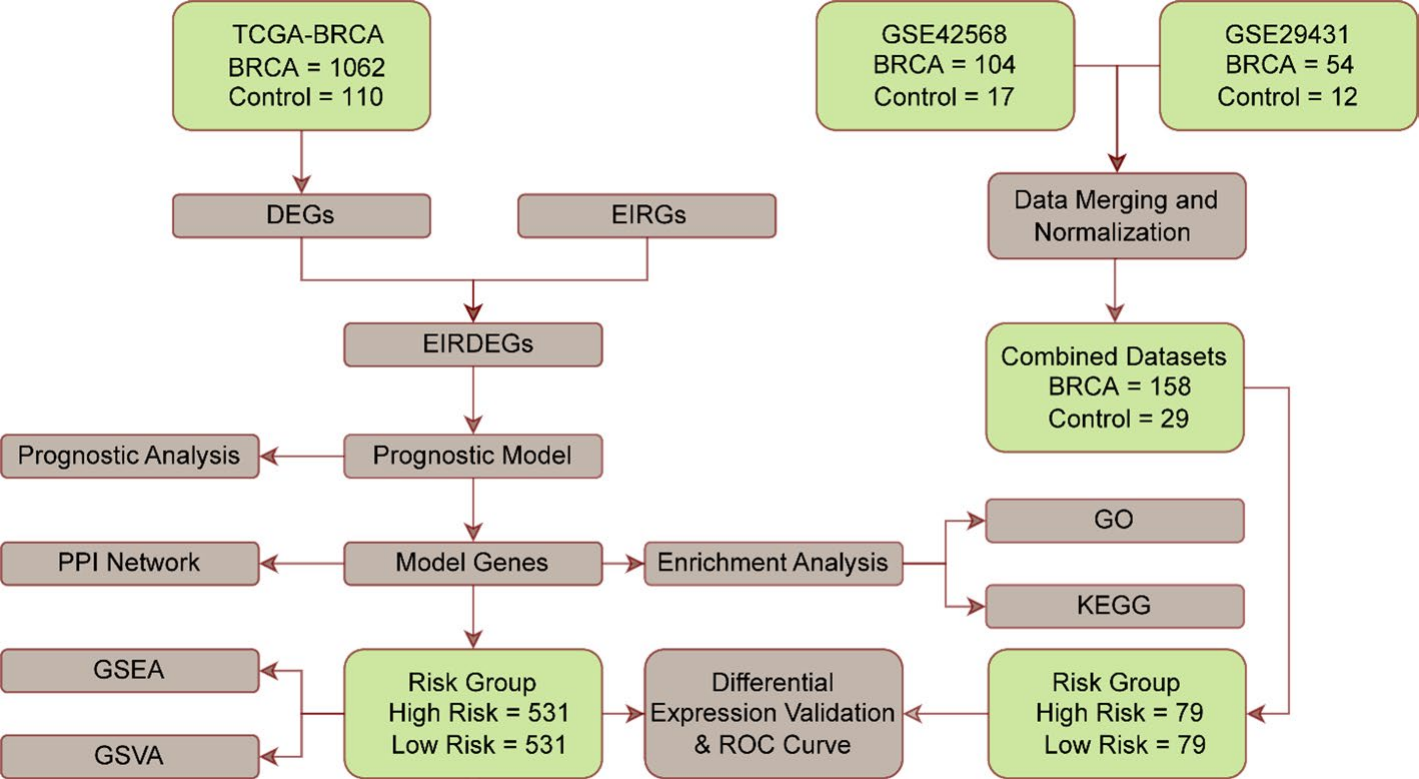
Flow chart for the analysis of EIRDEGs

The process of integrative analysis of EIRDEGs is shown in Fig. 1.

### BRCA-related efferocytosis- and invasion-related DEGs

**Fig. 2:**
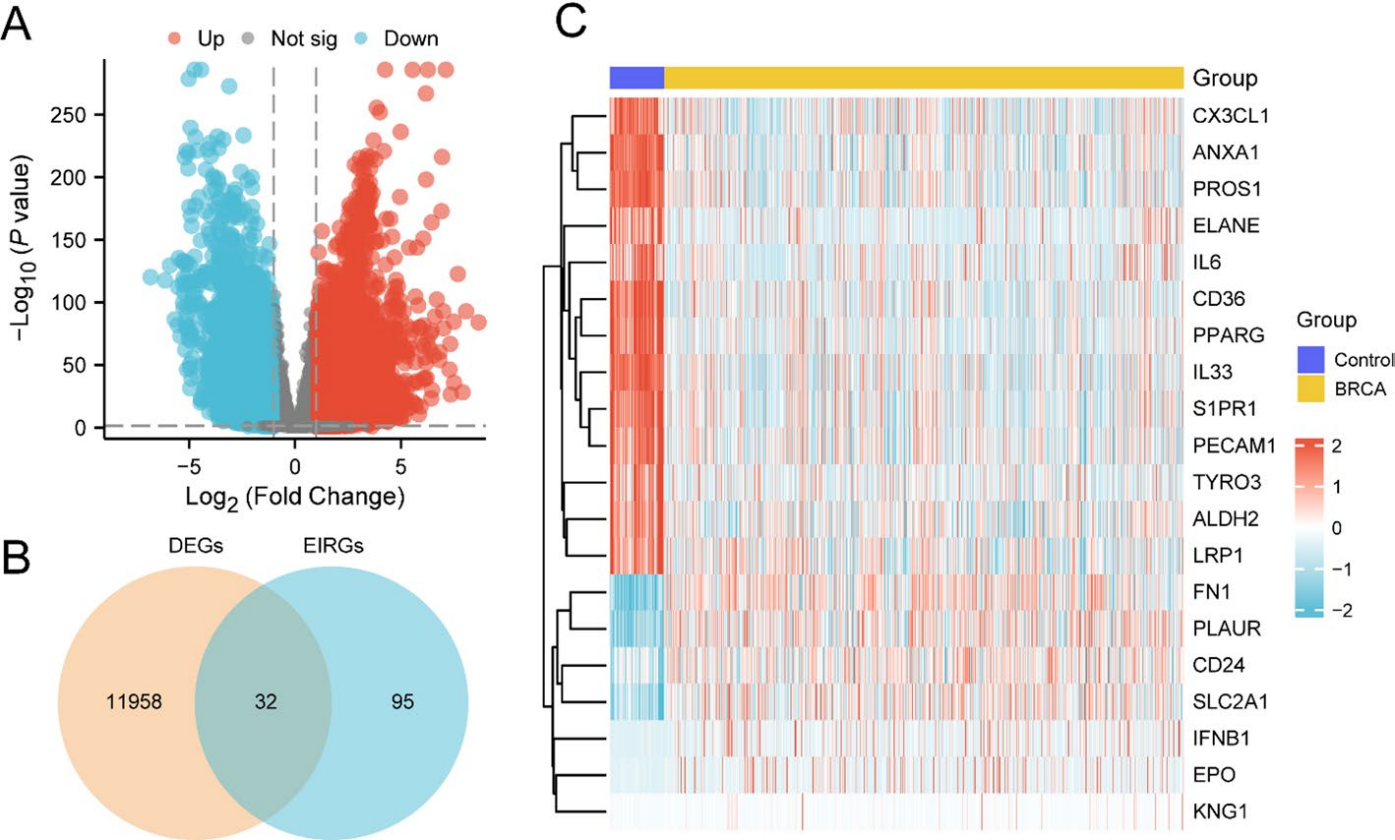
Differential Gene Expression Analysis. **A** Volcano plot of DEGs analysis between breast cancer (BRCA) cohort and Control (Control) cohort in the Breast Cancer Dataset (TCGA-BRCA). **B** DEG and EIRGs in the Breast Cancer dataset (TCGA-BRCA), Venn diagram of all genes in the Combined GEO Datasets. **C** Heat map of EIRDEGs in the breast cancer dataset (TCGA-BRCA). In the heat map grouping, blue is the Control (Control) cohort, and yellow is the BRCA cohort. In the heat map, red denotes elevated expression, and blue indicates reduced expression

Subsequently, the BRCA dataset (TCGA-BRCA) was categorized into BRCA and Control cohorts. Gene expression distinctions between these cohorts were examined. This investigation identified two DEG collections: 7,860 DEGs satisfied the parameters of|logFC| >1 and p-value < 0.05. Within these, 4,130 genes exhibited increased expression (logFC > 1 and p-value < 0.05), while 11,990 genes showed decreased expression (logFC < −1 and p-value < 0.05). The findings were illustrated through a volcano plot (Fig. 2A).

To detect EIRDEGs, all DEGs exhibiting|logFC| >1 and p-value < 0.05 were cross-referenced with EIRGs. A Venn diagram (Fig. 2B) was plotted to compare the DEGs with the EIRGs in the Combined GEO Datasets, revealing 32 EIRDEGs. Details regarding these genes appear in Table S2. Following this, the expression differences of the EIRDEGs in the TCGA-BRCA dataset were examined, and a heatmap was developed to visually present the outcomes (Fig. 2C).

### Construction of BRCA PRM

**Fig. 3:**
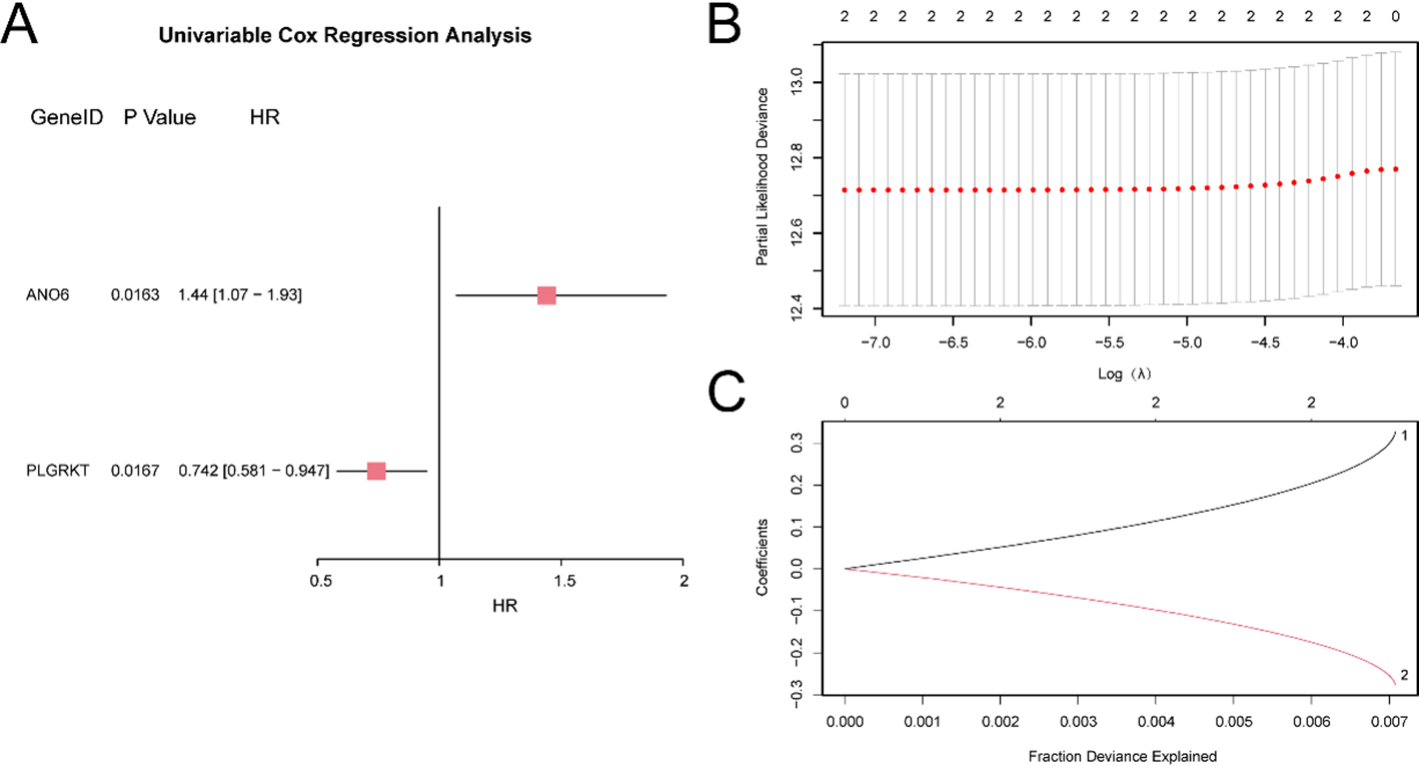
Cox Regression Analysis. **A** Forest Plot of 2 effemination and invasion associated differentially expressed genes (EIRDEGs) in univariate Cox regression model. **B**,** C** PRM plot (**B**) and variable trajectory plot (**C**) from the LASSO regression model

For BRCA PRM development, univariate Cox regression analysis was executed on the 32 EIRDEGs. Variables with a p-value < 0.05 in the univariate analysis were visualized utilizing a Forest plot (Fig. 3A). The results identified two statistically significant EIRDEGs (p-value < 0.05), denoted as *ANO6* and *PLGRKT*. To further examine the prognostic value of these genes in BRCA, LASSO regression analysis was conducted, and a LASSO regression model was developed. The LASSO model and variable trajectory plot (Fig. 3B-C) were generated for visualization. The LASSO regression model included two genes: *ANO6* and *PLGRKT*. The risk score was computed utilizing the following formula: risk score = *ANO6* × (0.328) + *PLGRKT* × (− 0.277).

### Prognostic analysis of BRCA PRM

**Fig. 4:**
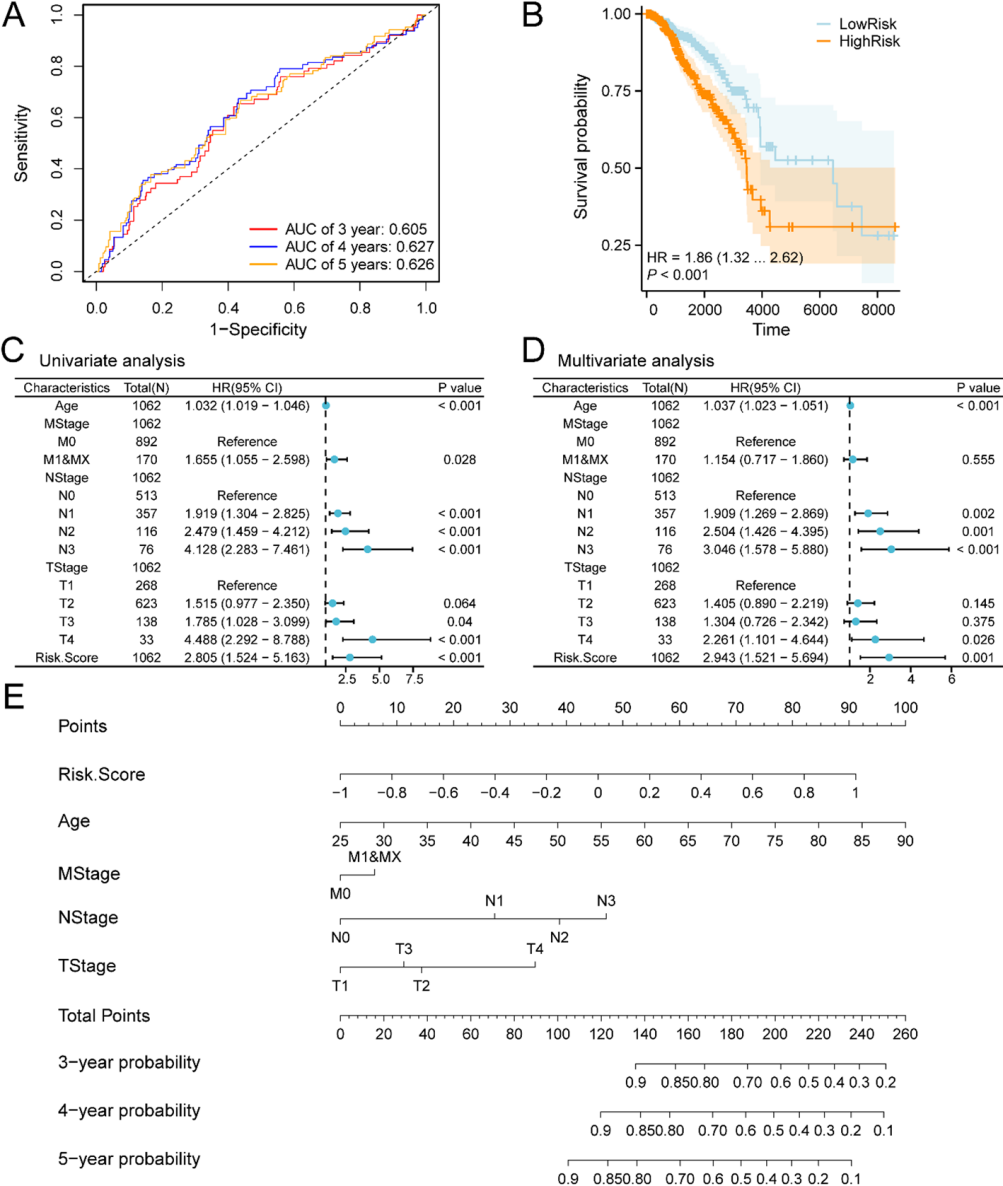
Prognostic analysis. **A** Temporal ROC curves for BRCA specimens in the breast cancer dataset (TCGA-BRCA). **B** Prognostic KM curves comparing high and low RiskScore cohorts and OS of BRCA specimens. **C**,** D** Forest Plot depicting RiskScore and clinical parameters in univariate Cox regression model (**C**) and multivariate Cox regression model (**D**). **E** Nomogram illustrating RiskScore and clinical parameters in univariate and multivariate Cox regression model. AUC values exceeding 0.5 suggest molecular expression’s inclination toward event occurrence. AUC values approaching 1 indicate enhanced diagnostic effectiveness, while values between 0.5–0.7 demonstrate limited precision. P values below 0.01 were deemed highly statistically significant

Time-dependent ROC curves (Fig. 4A) were generated for BRCA specimens within the TCGA-BRCA dataset. Findings demonstrated that the BRCA PRM displayed minimal precision (0.7 > AUC > 0.5) at 3, 4, and 5 years. Furthermore, KM curve examination (Fig. 4B) utilizing median OS categorization for BRCA specimens in the risk score-Combined Breast Cancer Dataset (TCGA-BRCA) exhibited substantial statistical distinctions between elevated and reduced risk categories (*p* < 0.01).

Following this, singular Cox regression examination incorporated risk metrics and clinical indicators to evaluate their overall survival (OS) prognostic significance in BRCA specimens. Parameters yielding p-values < 0.10 underwent multiple Cox regression examinations. Both singular and multiple Cox regression outcomes were illustrated through Forest plots (Fig. 4C, D) and detailed in Table 3. Multiple Cox regression examinations identified risk metrics, Age, NStage subcategories (N1, N2, and N3), and TStage subcategory (T4) as substantially significant indicators (*p* < 0.01).

**Table 3:** Results of Cox analysis

Characteristics	Total(*N*)	Univariate analysis		Multivariate analysis	
		HR (95% CI)	*P* value	HR (95% CI)	*P* value
Age	1062	1.032 (1.019–1.046)	< 0.001	1.037 (1.023–1.051)	< 0.001
MStage	1062				
M0	892	Reference		Reference	
M1&MX	170	1.655 (1.055–2.598)	0.028	1.154 (0.717–1.860)	0.555
NStage	1062				
N0	513	Reference		Reference	
N1	357	1.919 (1.304–2.825)	< 0.001	1.909 (1.269–2.869)	0.002
N2	116	2.479 (1.459–4.212)	< 0.001	2.504 (1.426–4.395)	0.001
N3	76	4.128 (2.283–7.461)	< 0.001	3.046 (1.578–5.880)	< 0.001
TStage	1062				
T1	268	Reference		Reference	
T2	623	1.515 (0.977–2.350)	0.064	1.405 (0.890–2.219)	0.145
T3	138	1.785 (1.028–3.099)	0.040	1.304 (0.726–2.342)	0.375
T4	33	4.488 (2.292–8.788)	< 0.001	2.261 (1.101–4.644)	0.026
Risk.Score	1062	2.805 (1.524–5.163)	< 0.001	2.943 (1.521–5.694)	0.001

*HR* hazard ratio

To enhance understanding of the risk model’s prognostic significance in BRCA, a Nomogram was developed incorporating both univariate and multivariate Cox regression analytical outcomes. This framework demonstrates the correlation between risk scores and four clinical parameters in BRCA specimens (Fig. 4E). The assessment indicated the prognostic significance of risk score and Age substantially exceeded other clinical parameters. In contrast, MStage, NStage, and TStage exhibited considerably lower utility in the PRM compared to the risk score.

**Fig. 5:**
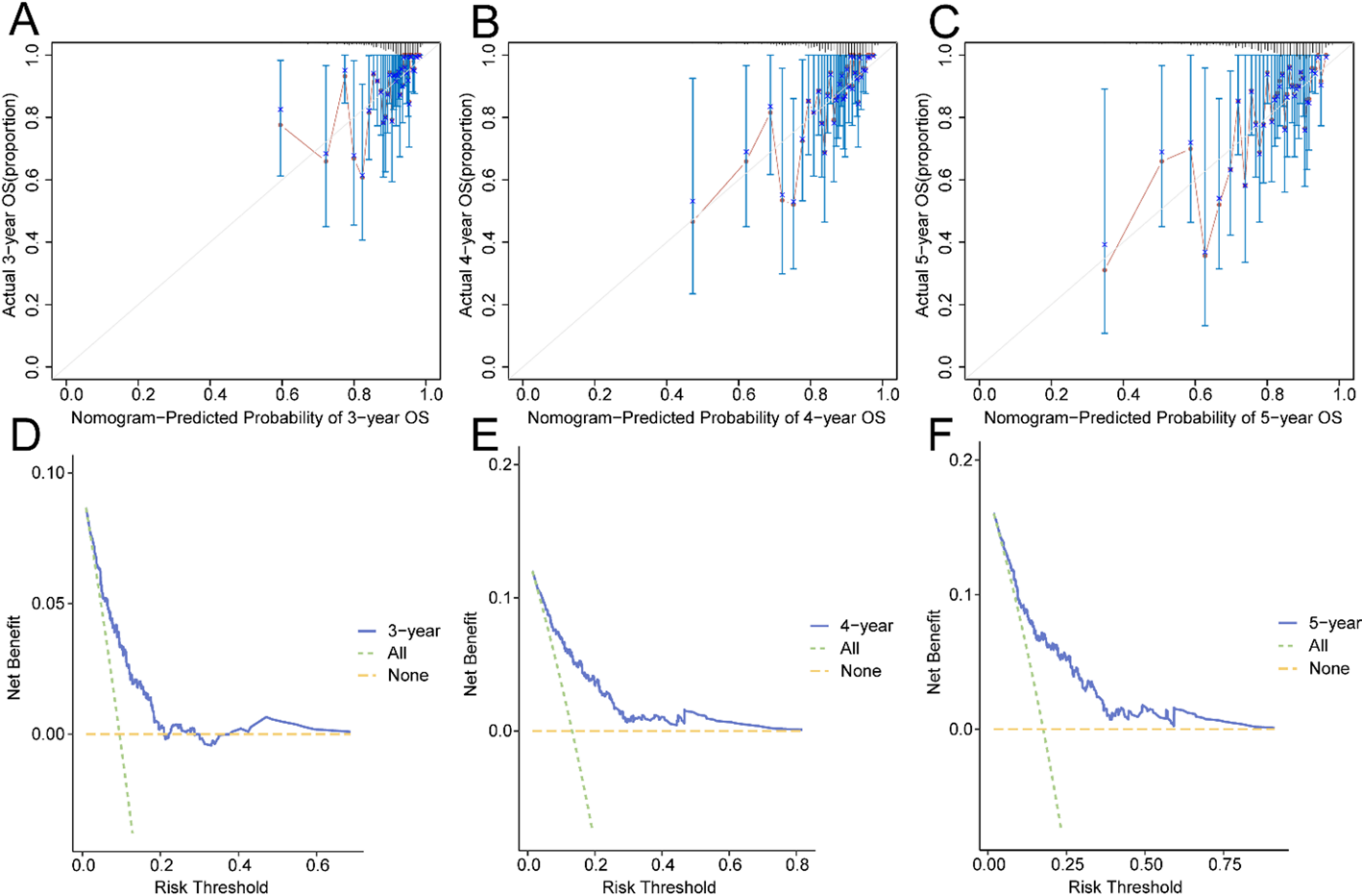
Prognostic Analysis. **A–C** 3-year (**A**), 4-year (**B**), and 5-year (**C**) Calibration curves of the BRCA PRM. **D–F** Prognosis of BRCA risk model 3 years (**D**), 4 years (**E**), 5 years (**F**) decision curve analysis (DCA)

Additionally, a calibration analysis was conducted for the 3-year, 4-year, and 5-year prognosis of the BRCA risk model, with Calibration Curves plotted (Fig. 5A–C). The horizontal axis of the Calibration Curve depicts model-predicted survival likelihood, while the vertical axis shows the actual survival probability. The lines representing the predicted survival probabilities at different time points closely aligned with the ideal gray line, indicating a better prediction at these time points. The outcomes demonstrated that the 5-year prediction had the best clinical performance for the BRCA risk model. Finally, DCA was utilized to examine the clinical effectiveness of the PRM at 3 years, 4 years, and 5 years (Fig. 5D–F). When the model’s line remained consistently above both the “All Positive” and “All Negative” lines within a certain range, a larger range was associated with a greater net benefit, reflecting better model performance. The analysis revealed that the LASSO regression model’s clinical prediction efficacy ranked: 5 years > 4 years > 3 years.

### GO and KEGG enrichment analyses

**Fig. 6:**
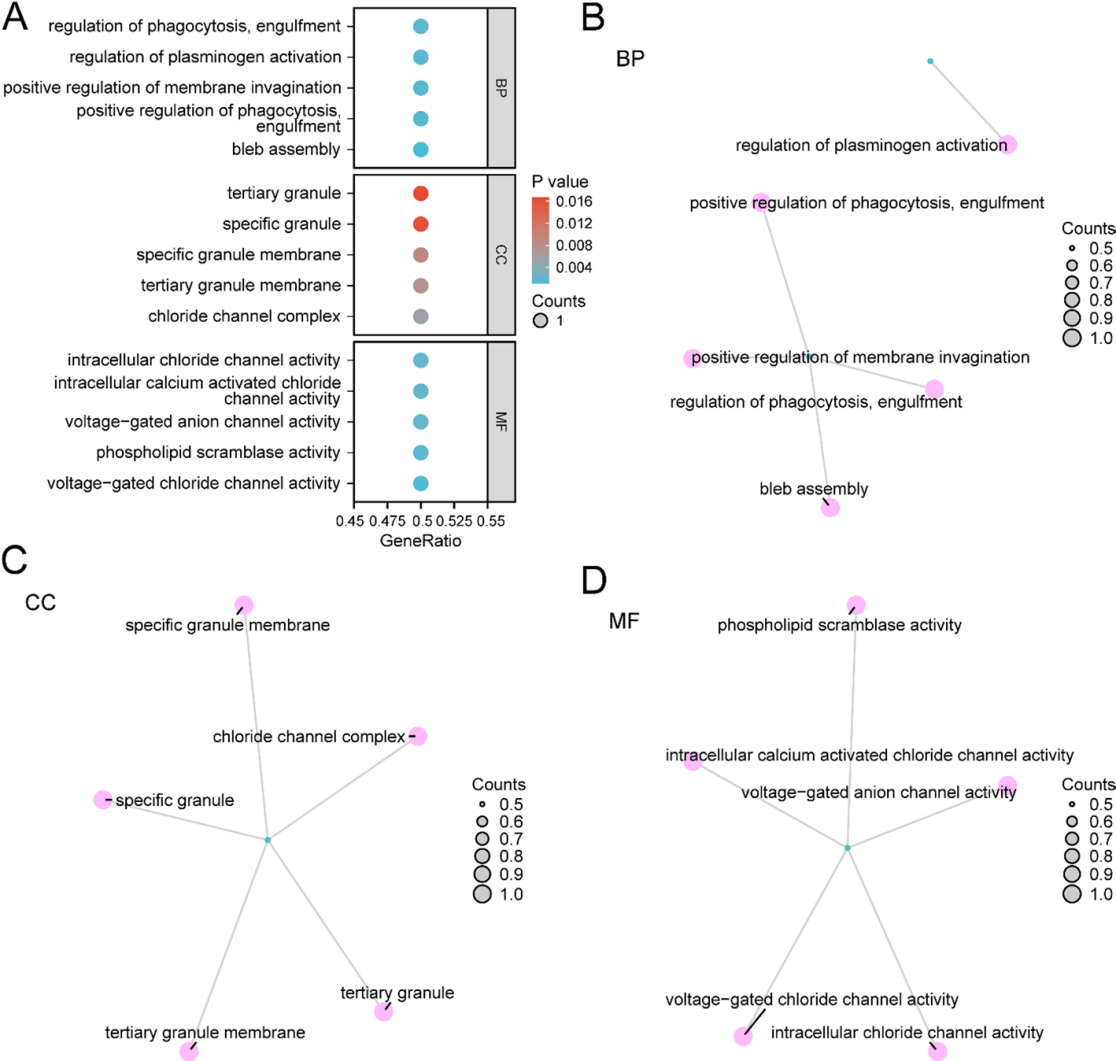
GO enrichment analysis for model genes. **A** The scatter visualization of GO enrichment analysis findings for Model Genes illustrated: BP, CC, MF, with GO terms on the vertical axis. **B–D** Model Genes (BP (**B**), CC (**C**), MF (**D**)) gene ontology (GO) enrichment analysis network illustration. Pink vertices represent entries, blue vertices indicate molecules, while connections demonstrate entry-molecule relationships. Within the scatter plot, circle dimensions reflect gene quantities, while color gradients indicate p-value magnitude. Deeper blue signifies lower p-values, while redder hues represent larger p-values. GO enrichment analysis employed a p value < 0.05 selection criterion

GO and KEGG pathway enrichment analyses were executed to further investigate the relationships between BPs, CCs, MFs, and biological pathways (KEGG) for the two Model Genes in BRCA. The detailed outcomes from the GO and KEGG enrichment analyses are depicted in Table 4. The examination demonstrated that the two Model Genes in BRCA exhibited enrichment predominantly in BPs encompassing bleb assembly, positive modulation of phagocytosis, positive modulation of membrane invagination, modulation of plasminogen activation, and phagocytosis. Regarding cellular components, the Model Genes exhibited enrichment in activities such as voltage-gated chloride channel activity, phospholipid scramblase activity, voltage-gated anion channel activity, and intracellular calcium-activated chloride channel activity. Regarding molecular functions, they were associated with the chloride channel complex, tertiary granule membrane, and specific granule membrane. Notably, no significant KEGG pathway enrichment was observed in this analysis. Bubble plots illustrated the GO enrichment analysis outcomes (Fig. 6A).

**Table 4:** Result of GO and KEGG enrichment analysis for model genes

ONTOLOGY	ID	Description	GeneRatio	BgRatio	pvalue	*p*.adjust	qvalue
BP	GO:0032060	bleb assembly	1/2	10/18,800	0.001063575	0.018172831	0.000164908
BP	GO:0060100	Positive regulation of phagocytosis, engulfment	1/2	13/18,800	0.001382537	0.018172831	0.000164908
BP	GO:1,905,155	Positive regulation of membrane invagination	1/2	13/18,800	0.001382537	0.018172831	0.000164908
BP	GO:0010755	Regulation of plasminogen activation	1/2	15/18,800	0.00159515	0.018172831	0.000164908
BP	GO:0060099	Regulation of phagocytosis, engulfment	1/2	15/18,800	0.00159515	0.018172831	0.000164908
MF	GO:0005247	Voltage-gated chloride channel activity	1/2	11/18,410	0.001194678	0.008600047	0.000822971
MF	GO:0017128	Phospholipid scramblase activity	1/2	14/18,410	0.001520376	0.008600047	0.000822971
MF	GO:0008308	Voltage-gated anion channel activity	1/2	17/18,410	0.00184602	0.008600047	0.000822971
MF	GO:0005229	Intracellular calcium activated chloride channel activity	1/2	18/18,410	0.001954556	0.008600047	0.000822971
MF	GO:0061778	Intracellular chloride channel activity	1/2	18/18,410	0.001954556	0.008600047	0.000822971
CC	GO:0034707	Chloride channel complex	1/2	52/19,594	0.005300839	0.027801673	
CC	GO:0070821	Tertiary granule membrane	1/2	73/19,594	0.00743757	0.027801673	
CC	GO:0035579	Specific granule membrane	1/2	91/19,594	0.009267224	0.027801673	
CC	GO:0042581	Specific granule	1/2	160/19,594	0.016265264	0.030006336	
CC	GO:0070820	Tertiary granule	1/2	164/19,594	0.016670187	0.030006336	

*GO* gene ontology, *BP* biological process, *CC* cellular component, *MF* molecular function

Additionally, network maps for BPs, CCs, and MFs were created based on the GO enrichment analysis (Fig. 6B–D). These maps illustrate the relationships between the corresponding annotations and molecules, where nodes with larger entries represent a higher number of associated molecules.

### GSEA for high- and low-risk cohorts

**Fig. 7:**
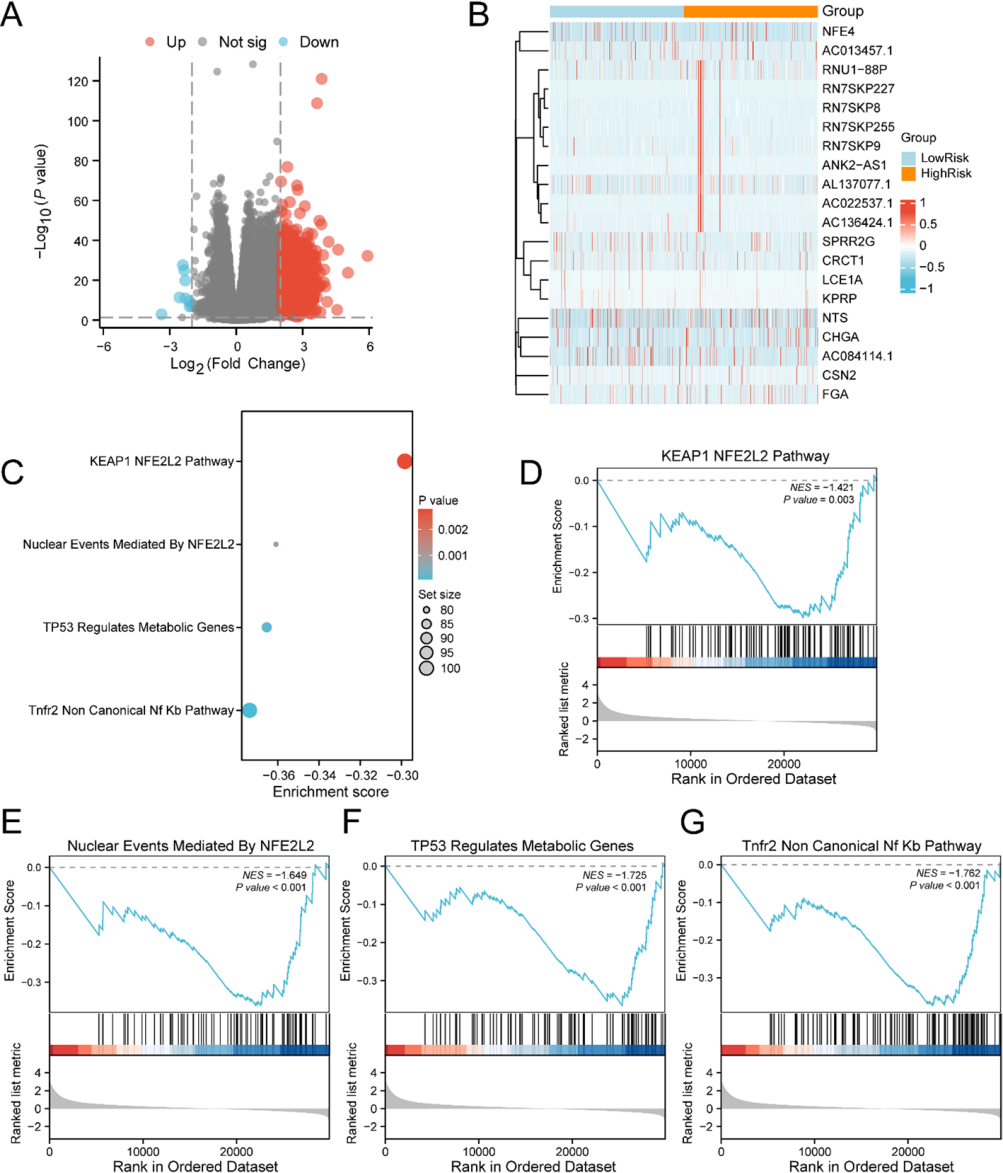
GSEA for Risk Cohort. **A** Volcano plot of DEGs analysis between High Risk cohort and Low Risk cohort in BRCA samples in the Breast Cancer Dataset (TCGA-BRCA). **B** logFC ranked Top10 up-regulated and Top10 down-regulated DEGs heatmap of BRCA samples in High Risk cohort and Low Risk cohort. **C** Display of 4 biological function bubble plots from GSEA of Breast cancer dataset (TCGA-BRCA). **D–G** GSEA depicted that genes in the breast cancer dataset (TCGA-BRCA) were markedly enriched in KEAP1 NFE2L2 Pathway (**D**), Nuclear Events Mediated By NFE2L2 (**E**), KEAP1 NFE2L2 pathway (**D**), and nuclear events mediated by NFE2L2 (**E**). *TP53* Regulates Metabolic Genes (**F**), Tnfr2 non-canonical NF-κB Pathway (**G**). The heatmap utilizes orange for High Risk cohort and blue for Low Risk cohort designation. Expression levels are depicted in red (high) and blue (low). Bubble dimensions correspond to enriched gene quantities, while bubble coloration reflects P-value magnitude. Blue shades indicate smaller P-values, while redder shades signify larger values. GSEA employed a p value < 0.05 threshold

To perform a differential analysis of BRCA specimens in the TCGA-BRCA dataset, BRCA samples were stratified into high-risk and low-risk cohorts utilizing the median risk score from the BRCA Risk model. DEGs between these cohorts were ascertained, with the following results: 1,138 DEGs satisfied the parameters of|logFC| >2 and p-value < 0.05. Within this set, 1129 genes exhibited increased expression (logFC > 2 and p-value < 0.05), while 9 genes showed decreased expression (logFC < −2 and p-value < 0.05). A volcano plot illustrated these comparative findings (Fig. 7A). Furthermore, the heatmap displaying the logFC of the leading 10 increased and decreased genes (Fig. 7B).

To investigate gene expression influence on BRCA, GSEA was conducted on TCGA-BRCA BRCA specimens to explore connections between gene expression and various BPs, cellular components, and molecular functions (Fig. 7C). GSEA findings are listed in Table 5. The investigation demonstrated significant gene enrichment in multiple essential pathways, encompassing the KEAP1-NFE2L2 pathway (Fig. 7D), Nuclear Events Mediated by NFE2L2 (Fig. 7E), NFE2L2 pathway (Fig. 7E), *TP53* Regulation of Metabolic Genes (Fig. 7F), and the TNFR2 non-canonical NF-κB pathway (Fig. 7G), among others, highlighting their potential roles in BRCA biology and signaling pathways.

**Table 5:** Results of GSEA for risk group

ID	setSize	Enrichment score	NES	pvalue	*p*.adjust	qvalue
REACTOME_KEAP1_NFE2L2_PATHWAY	102	− 0.298389771	− 1.421097896	0.002794319	0.046906014	0.042660272
REACTOME_NUCLEAR_EVENTS_MEDIATED_BY_NFE2L2	79	− 0.360936382	− 1.648618016	0.000749649	0.016587691	0.01508624
REACTOME_TNFR2_NON_CANONICAL_NF_KB_PATHWAY	100	− 0.373704464	− 1.762146174	7.32298E−05	0.002418303	0.002199408
REACTOME_DECTIN_1_MEDIATED_NONCANONICAL_NF_KB_SIGNALING	62	− 0.502013035	− 2.236002913	1.14814E−06	7.55293E−05	6.86927E−05
REACTOME_P130CAS_LINKAGE_TO_MAPK_SIGNALING_FOR_INTEGRINS	15	0.739268014	1.707487623	0.003211544	0.051767538	0.047081751
REACTOME_GRB2_SOS_PROVIDES_LINKAGE_TO_MAPK_SIGNALING_FOR_INTEGRINS	15	0.72338701	1.670807261	0.005630803	0.077431498	0.070422714
REACTOME_SIGNALING_BY_NOTCH4	82	− 0.360259982	− 1.702810769	0.000448407	0.010914236	0.009926323
REACTOME_NEGATIVE_REGULATION_OF_NOTCH4_SIGNALING	54	− 0.54947764	− 2.380969134	6.7347E−07	4.82125E−05	4.38485E−05
REACTOME_HEDGEHOG_OFF_STATE	112	− 0.335724046	− 1.618455669	0.000190314	0.005514576	0.005015419
REACTOME_HEDGEHOG_LIGAND_BIOGENESIS	65	− 0.473871235	− 2.130389985	1.42935E−05	0.000669044	0.000608485
REACTOME_TP53_REGULATES_METABOLIC_GENES	84	− 0.36537644	− 1.72450198	0.000185575	0.005493936	0.004996647
REACTOME_KEAP1_NFE2L2_PATHWAY	102	− 0.298389771	− 1.421097896	0.002794319	0.046906014	0.042660272
REACTOME_NUCLEAR_EVENTS_MEDIATED_BY_NFE2L2	79	− 0.360936382	− 1.648618016	0.000749649	0.016587691	0.01508624

### GSVA for high- and low-risk cohorts

**Fig. 8:**
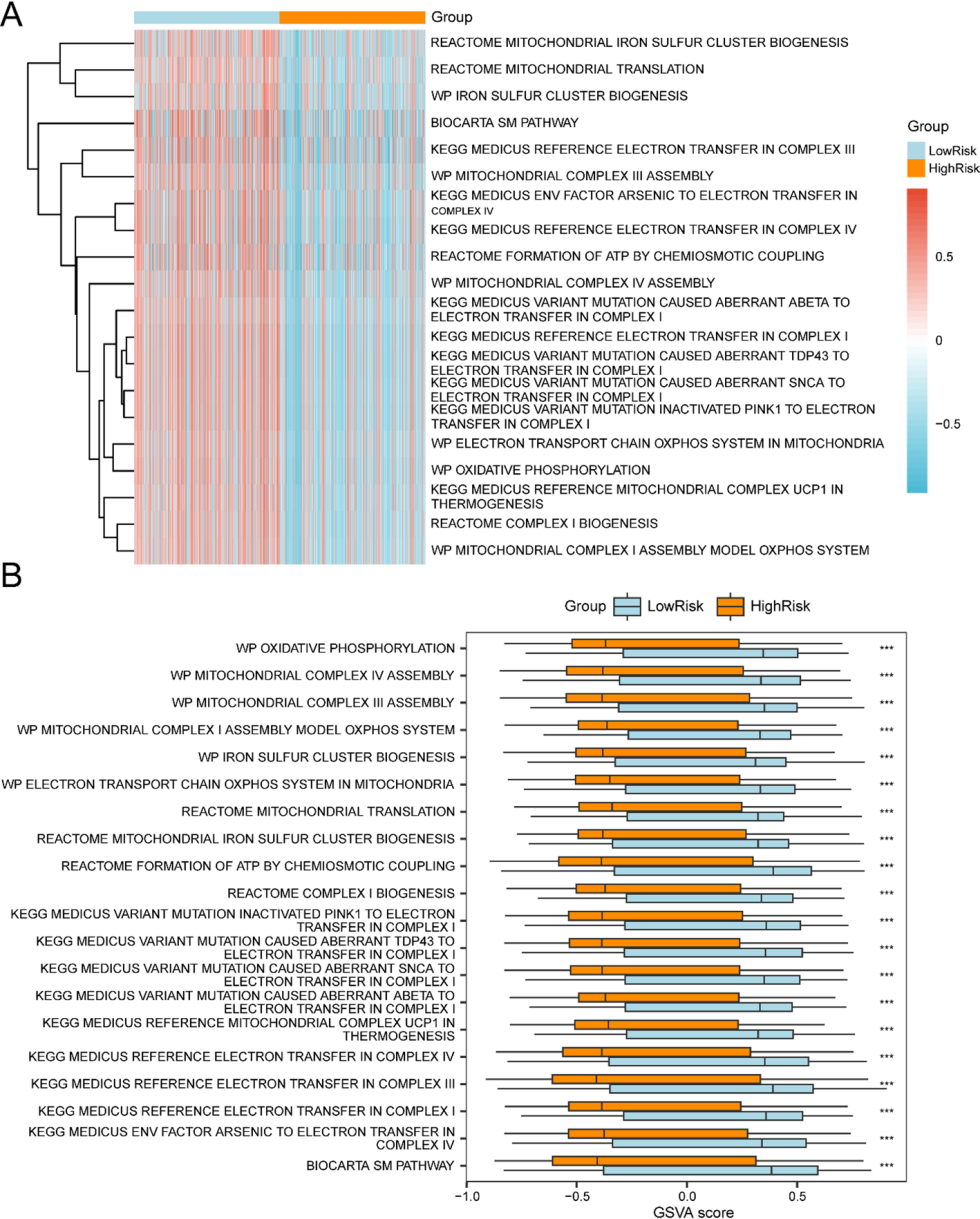
GSVA Analysis. **A**,** B** Heat map (**A**) and comparative visualization (**B**) of gene set variant analysis (GSVA) findings between High Risk and Low Risk cohorts from TCGA-BRCA dataset specimens. *** denotes p value < 0.001, indicating substantial statistical significance. Orange indicates the High Risk cohort while blue signifies the Low Risk cohort. The selection parameters for gene set variation analysis (GSVA) utilized p-value < 0.05, with Benjamini-Hochberg (BH) adjustment for p-value correction. In the heat map, blue indicates minimal enrichment while red signifies substantial enrichment

To examine distinctions between elevated and reduced risk categories in TCGA-BRCA dataset specimens, GSVA implementation utilized the c2.cp.v2023.2.Hs.symbols.gmt gene collection. The examination encompassed all BRCA dataset genes, with findings presented in Table 6. Post-GSVA identification focused on the top 20 pathways exhibiting p-value < 0.05 and maximum absolute logFC values. These pathways underwent differential expression analysis between elevated and reduced risk categories, with visualization through a heatmap (Fig. 8A). The heatmap effectively demonstrates pathway expression variations between risk categories. For validation purposes, Mann-Whitney U testing provided statistical verification, with findings depicted in a group comparison chart (Fig. 8B). The analysis revealed statistically significant pathways, confirming expression differences between high-risk and low-risk cohorts(p-values < 0.05).

**Table 6:** Results of GSVA for risk group

Pathway	logFC	AveExpr	t	*P*.Value	adj.*P*.Val	B
KEGG MEDICUS VARIANT MUTATION CAUSED ABERRANT TDP43 TO ELECTRON TRANSFER IN COMPLEX I	− 0.649960922	− 0.024804031	− 16.06094387	3.16E−52	2.83E−49	107.9126842
KEGG MEDICUS REFERENCE ELECTRON TRANSFER IN COMPLEX I	− 0.647630454	− 0.024581824	− 15.92686555	1.80E−51	9.21E−49	106.1863574
KEGG MEDICUS VARIANT MUTATION CAUSED ABERRANT SNCA TO ELECTRON TRANSFER IN COMPLEX I	− 0.640529328	− 0.024078342	− 16.04881745	3.70E−52	2.83E−49	107.7561845
KEGG MEDICUS VARIANT MUTATION INACTIVATED PINK1 TO ELECTRON TRANSFER IN COMPLEX I	− 0.637070915	− 0.023625245	− 15.86016317	4.27E−51	1.87E−48	105.3308615
WP MITOCHONDRIAL COMPLEX IV ASSEMBLY	− 0.619678527	− 0.035739229	− 15.31653772	4.45E−48	1.36E−45	98.44287446
REACTOME COMPLEX I BIOGENESIS	− 0.617311196	− 0.026612511	− 16.2653475	2.18E−53	3.35E−50	110.5614932
WP MITOCHONDRIAL COMPLEX I ASSEMBLY MODEL OXPHOS SYSTEM	− 0.616000229	− 0.028324822	− 16.56785807	4.04E−55	1.24E−51	114.5186333
WP OXIDATIVE PHOSPHORYLATION	− 0.614585594	− 0.028012153	− 15.52718655	3.07E−49	1.04E−46	101.0938647
KEGG MEDICUS REFERENCE MITOCHONDRIAL COMPLEX UCP1 IN THERMOGENESIS	− 0.611982814	− 0.026983059	− 15.97762845	9.33E−52	5.72E−49	106.8389073
WP MITOCHONDRIAL COMPLEX III ASSEMBLY	− 0.611972531	− 0.031458591	− 14.5970323	3.45E−44	7.06E−42	89.56616686
KEGG MEDICUS REFERENCE ELECTRON TRANSFER IN COMPLEX III	− 0.601838416	− 0.02861327	− 12.50557898	1.29E−33	1.02E−31	65.45926497
BIOCARTA SM PATHWAY	− 0.601521532	− 0.034550359	− 12.40314129	3.97E−33	2.83E−31	64.34818533
KEGG MEDICUS VARIANT MUTATION CAUSED ABERRANT ABETA TO ELECTRON TRANSFER IN COMPLEX I	− 0.60038374	− 0.027739762	− 15.83513529	5.90E−51	2.26E−48	105.0104396
REACTOME FORMATION OF ATP BY CHEMIOSMOTIC COUPLING	− 0.59626833	− 0.028984448	− 13.02622277	3.87E−36	3.59E−34	71.21067852
KEGG MEDICUS REFERENCE ELECTRON TRANSFER IN COMPLEX IV	− 0.589107255	− 0.043329756	− 13.16625069	7.87E−37	8.32E−35	72.78675572
WP ELECTRON TRANSPORT CHAIN OXPHOS SYSTEM IN MITOCHONDRIA	− 0.577611407	− 0.025009271	− 14.89998109	8.23E−46	1.80E−43	93.26953991
WP IRON SULFUR CLUSTER BIOGENESIS	− 0.573733757	− 0.049065231	− 14.58080515	4.21E−44	8.07E−42	89.36923004
REACTOME MITOCHONDRIAL IRON SULFUR CLUSTER BIOGENESIS	− 0.570711009	− 0.047993282	− 14.44558565	2.20E−43	3.54E− 41	87.73389703
REACTOME MITOCHONDRIAL TRANSLATION	− 0.566449945	− 0.021626643	− 15.20431715	1.83E−47	5.11E−45	97.04007782
KEGG MEDICUS ENV FACTOR ARSENIC TO ELECTRON TRANSFER IN COMPLEX IV	− 0.563809073	− 0.032644963	− 13.05372466	2.83E−36	2.71E−34	71.51925948

### PPI network

**Fig. 9:**
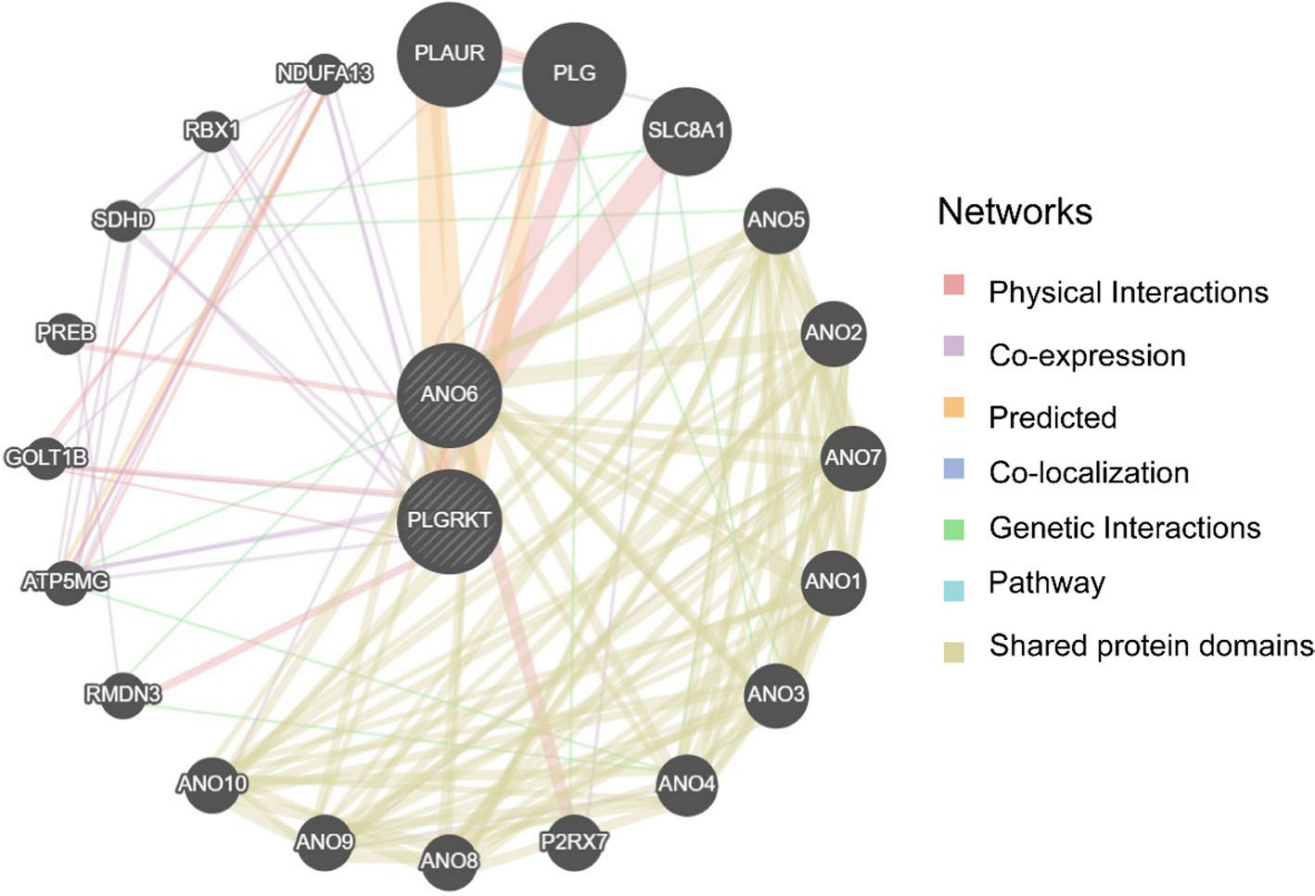
PPI Network Analysis. GeneMANIA website forecasts interaction networks of functionally comparable Genes relative to two Model Genes. Within the illustration, circles represent Model Genes and their functionally similar counterparts, while line colors signify their interconnected functional relationships

Furthermore, a PPI network of the two Model Genes and their functionally similar genes was constructed utilizing the GeneMANIA website (Fig. 9). The network visually represents the co-expression links between the two Model Genes and their similar genes, with different colors in the lines indicating different types of relationships, such as co-expression or shared protein domains. The network revealed that the two Model Genes were functionally related to 20 similar proteins, which are detailed in Table S3.

### Differential expression verification and ROC curve analysis between high- and low-risk cohorts

**Fig. 10:**
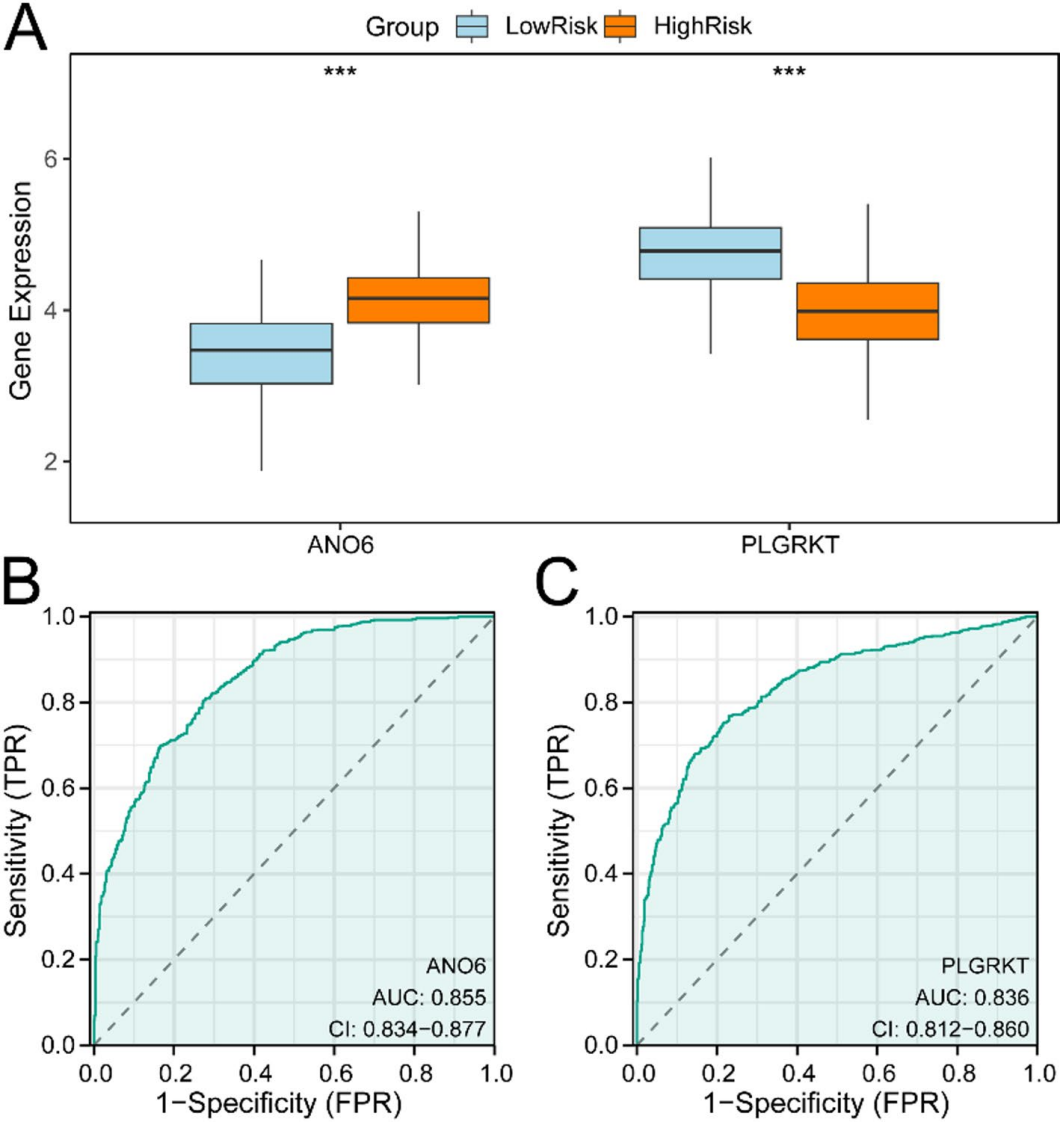
Differential Expression Validation and ROC Curve Analysis. **A** Model Genes comparison visualization between High Risk and Low Risk cohorts within BRCA specimens from TCGA-BRCA dataset. **B**,** C** Model Genes *ANO6* (**B**), *PLGRKT* (**C**) ROC analysis in TCGA-BRCA dataset specimens. *** denotes p value < 0.001, indicating substantial statistical significance. AUC exceeding 0.5 suggests molecular expression’s propensity toward event occurrence. AUC proximity to 1 indicates enhanced diagnostic efficacy, with values between 0.7–0.9 demonstrating notable precision. Orange designates High Risk cohort while light blue represents Low Risk cohort. TPR, True Positive Rate; FPR, False Positive Rate

TCGA-BRCA dataset specimens were stratified into high-risk and low-risk cohorts utilizing median risk values from the BRCA prognostic framework. To investigate model gene expression variations in these specimens, a comparative evaluation (Fig. 10A) was implemented, revealing substantial distinctions in expression patterns of two model genes, A and B, across high-risk and low-risk cohorts. The investigation confirmed that model genes *ANO6* and *PLGRKT* exhibited notably distinct expression patterns between these cohorts (*p* < 0.001). Additionally, ROC analyses were conducted to evaluate model gene predictive capabilities (Fig. 10B, C). These analyses demonstrated that model genes *ANO6* and *PLGRKT* expression levels in the TCGA-BRCA dataset effectively distinguished between high-risk and low-risk cohorts, yielding AUC values between 0.7 and 0.9, indicating robust classification effectiveness.

### Differential expression verification and ROC curve analysis of the integrated GEO dataset

**Fig. 11:**
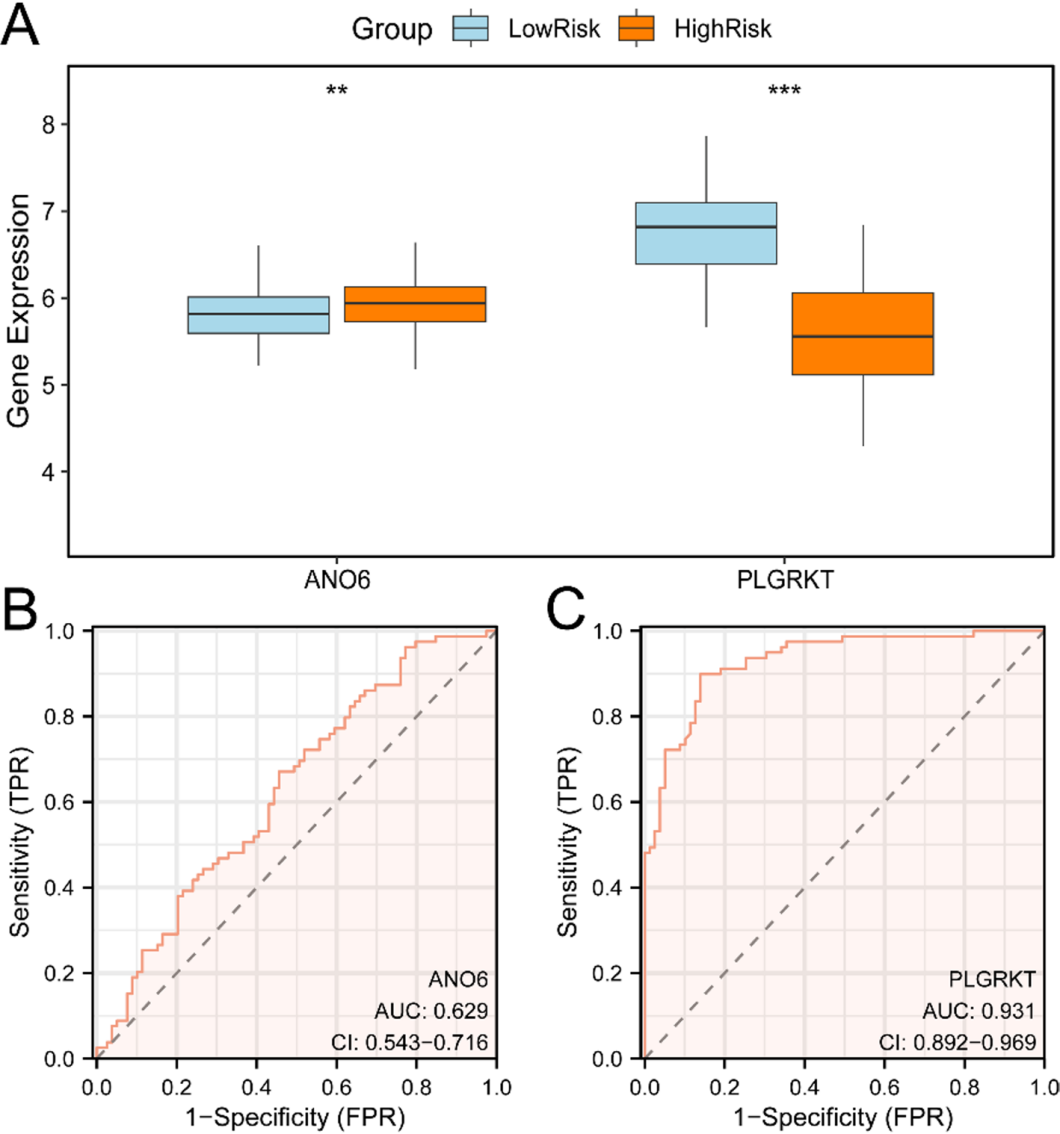
Differential Expression Validation and ROC Curve Analysis. **A** Model Gene comparison plots between High Risk and Low Risk categories in BRCA specimens from Combined GEO Datasets. **B**,** C** ROC analyses of Model Genes *ANO6* (**B**), *PLGRKT* (**C**) in BRCA specimens from integrated GEO Datasets (Combined Datasets). ** denotes p value < 0.01, indicating substantial statistical significance; *** represents p value < 0.001, demonstrating exceptional statistical significance. AUC values exceeding 0.5 suggest molecular expression’s promotion of events, with values approaching 1 indicating superior diagnostic outcomes. AUC demonstrates minimal precision from 0.5–0.7 and intermediate precision from 0.7–0.9. Orange indicates the High Risk category, while blue represents the Low Risk category

Risk scores for BRCA specimens from consolidated GEO datasets utilized risk coefficients derived from the BRCA prognostic framework. Specimens were classified into high-risk and low-risk categories utilizing median risk expression values. A comparative examination (Fig. 11A) of model gene expression patterns revealed notable variations between high-risk and low-risk categories. The assessment demonstrated that model genes *ANO6* and *PLGRKT* exhibited substantially elevated expression in high-risk specimens compared to low-risk counterparts (*p* < 0.001). The pROC R package facilitated ROC analysis to assess the predictive capabilities of genes *ANO6* and *PLGRKT* (Fig. 11B, C). Results indicated that *ANO6* expression in the consolidated GEO dataset showed minimal classification precision (0.5 ≤ AUC < 0.7) in distinguishing high-risk and low-risk categories, while *PLGRKT* demonstrated intermediate classification precision (0.7 ≤ AUC < 0.9).

## Discussion

BRCA represents a primary source of cancer-related morbidity and mortality among females globally. It is a heterogeneous disease comprising various subtypes, each with distinct biological and clinical characteristics. The high prevalence and substantial impact of BRCA on patients’ quality of life and survival underscore the critical need for ongoing research and improved therapeutic strategies.

By systematically integrating efferocytosis- and invasion-related genes, this study identified *ANO6* and *PLGRKT* as dual-process-driven prognostic biomarkers in breast cancer. The two-gene PRM, constructed via LASSO regression, demonstrated moderate predictive accuracy in the TCGA cohort (3-year AUC: 0.605; 5-year AUC: 0.626), with significant survival stratification between high- and low-risk groups (KM log-rank *P* < 0.001). External validation in merged GEO datasets confirmed the robustness of *PLGRKT* (AUC = 0.931), though *ANO6* exhibited reduced generalizability (AUC = 0.629), suggesting subtype-specific biological variability [46]. The antagonistic risk weights of *ANO6* (positive coefficient) and *PLGRKT* (negative coefficient) reflect their potential functional interplay in balancing tumor microenvironment dynamics, providing a novel molecular lens for deciphering breast cancer heterogeneity.

Functional enrichment analyses revealed that *ANO6* and *PLGRKT* converge on phagocytosis regulation and membrane remodeling. ANO6, a calcium-activated chloride channel with phospholipid scramblase activity, likely facilitates efferocytosis by exposing phosphatidylserine (PS) on the outer membrane—a critical “eat-me” signal for tumor-associated macrophages (TAMs) [47]. Concurrently, its ion flux regulation may promote cytoskeletal reorganization, enabling invasive pseudopod formation [48]. In contrast, *PLGRKT*, a plasminogen receptor, appears to inhibit ECM degradation by sequestering plasminogen from urokinase-mediated activation [49]. Collectively, high-risk BRCA is characterized by compromised antioxidant capacity, metabolic dysregulation, and suppressed inflammatory signaling, whose synergistic effects may accelerate malignant progression and poor clinical outcomes. Therapeutic strategies targeting NRF2 activation, restoring *TP53*-mediated metabolic control, or modulating NF-κB signaling could offer novel avenues for high-risk patient management [50].

The PPI network further linked *ANO6* to calcium signaling regulators (e.g., SLC4A1) and *PLGRKT* to integrin-mediated adhesion molecules (e.g., ITGB1) [48]. These interactions suggest a coordinated mechanism: *ANO6*-mediated ion dyshomeostasis primes early metastatic niche formation, while *PLGRKT* loss exacerbates ECM stiffness and inflammatory signaling, collectively fueling tumor progression [51]. This spatiotemporal synergy underscores the dual-process model’s biological plausibility, where efferocytosis evasion and stromal co-option operate as complementary hallmarks of aggressive disease.

Multivariate analysis identifies lymph node metastasis (N stage), risk score, age, and T4 tumors as independent prognostic factors in high-risk BRCA patients, while M stage and T2/T3 tumors showed no significant impact. These findings provide critical insights for refining risk stratification and guiding personalized therapeutic strategies. Decision curve analysis (DCA) confirmed its clinical utility, showing net benefit advantages over TNM staging at 5-year predictions. For early-stage patients lacking conventional high-risk features, the model could refine adjuvant therapy selection—high-risk subgroups may benefit from NF-κB inhibitors (e.g., bortezomib) or oxidative stress-targeting agents [50], as suggested by GSVA-identified pathway enrichments. Importantly, *PLGRKT*’s stable diagnostic performance across cohorts positions it as a priority biomarker for clinical assay development [52]. Unlike conventional single-mechanism models (e.g., MMP-based invasion signatures [48] or efferocytosis-focused markers like GAS6/MFG-E8 [47]), this study pioneers a dual-pathway integration strategy. While Oncotype DX^®^ and similar multi-gene panels achieve comparable accuracy [53], their “black-box” algorithms lack mechanistic interpretability. Here, *ANO6* and *PLGRKT* offer direct therapeutic targeting potential: *ANO6* inhibitors (e.g., CaCCinh-A01) could disrupt efferocytosis-immune crosstalk [54], whereas *PLGRKT* agonists might restore ECM homeostasis [49]. Compared to EMT-driven models (e.g., ZEB1/TWIST1 [48]), our PRM uniquely captures microenvironmental interplay, explaining its enhanced performance in HER2 + subtypes [55]. These advances align with emerging trends in precision oncology [56], where microenvironment-aware biomarkers are prioritized for combination therapy design.

In summary, this study revealed the clinical application value of efferocytosis- and invasion-related genes in the prognostic assessment of breast cancer through a multi-omics integration strategy, and provided a theoretical basis for the development of novel targeted therapeutic strategies. Subsequent studies can verify the function of key genes by CRISPR screening and other technologies, and explore their synergistic effects with existing therapeutic regimens (e.g., CDK4/6 inhibitors), to promote the development of precision therapy for breast cancer.

## Limitations of the study

The limitations of this study are fourfold: First, breast cancer is highly heterogeneous in terms of molecular characteristics, patient demographics, and geographic distribution. Although our study incorporated large-scale TCGA and GEO datasets with rigorous correction for batch effects, the model construction relied on retrospective public databases (TCGA/GEO), which are at risk of selection bias, especially since Asian populations accounted for less than 12% of the samples. This may affect the model’s generalization ability to non-European and American populations, limiting the generalizability to global populations. Second, the biological function validation of *ANO6*/*PLGRKT* is limited to bioinformatics speculation and lacks validation in organoid models or transgenic animal experiments. Wet-laboratory experiments could provide more direct evidence to support the biological mechanism of differential expression of genes associated with cell expulsion and invasion in BRCA. Third, GeneCards integrates gene-related information mainly from existing literature and databases. However, its screening results may be affected by current research bias and may overlook genes with low expression levels, insufficient investigation, or incomplete functional classification. Such limitations may exclude biologically important genes that have been implicated in the pathogenesis of breast cancer. Finally, the lack of clinical validation (e.g., prospective cohort studies or clinical trials) necessitates further confirmation of the predictive power of PRM in real-world settings.

Future research suggests in-depth exploration in four dimensions: (1) Database level: Implementation of a multi-database integration strategy using platforms such as MSigDB, STRING, and Reactome to expand the ethnic and geographic diversity of the study cohort, refine gene selection algorithms by network-based analyses, and enhance the robustness of the results by cross-platform validation methods. (2) Experimental validation level: Combining knockdown of *ANO6*/*PLGRKT* by CRISPR-Cas9 with 3D tumor sphere invasion assay and live cell imaging to dynamically resolve its effect on efferocytosis efficiency. (3) Clinical translation level: Prospective collection of samples from breast cancer patients (*n* > 200) treated with CDK4/6 inhibitors, and use of digital droplet PCR to monitor both efferocytosis efficiency and gene expression dynamics before and after treatment, and establishment of a prediction model for drug response. (4) Technological innovation level: Integration of spatial transcriptome (10x Visium) and multiple ion beam imaging (MIBI-TOF) to map the spatial expression of *ANO6*/*PLGRKT* at tumor-immune boundaries, and to reveal its mechanism of remodeling microenvironments that regulate immune escape.

## Conclusions

In conclusion, this investigation effectively distinguished differential efferocytosis- and invasion-associated genes in BRCA and established a PRM with substantial predictive capability. The framework exhibited robust precision and distinction in forecasting patient outcomes, confirmed through diverse statistical evaluations. FEAs demonstrated that the model-associated genes participate in essential BPs, cellular structures, and molecular activities, highlighting their significance in BRCA advancement. The PPI network illuminated the gene interconnections, offering an understanding of their combined influence on the condition. Subsequent investigations should emphasize clinical validation and laboratory experimentation to enhance the model’s practical medical application. Synthesizing these discoveries could support the advancement of individualized and efficient therapeutic approaches for BRCA patients.

## Supplementary Information

Supplementary Material 1

Supplementary Material 2

Supplementary Material 3

Supplementary Material 4

Supplementary Material 5

## Data Availability

The datasets generated for this study can be found in the TCGA-BRCA database from the TCGA portal (https://portal.gdc.cancer.gov/). Human gene information of TCGA-BRCA patients were downloaded from The GeneCards database (https://www.genecards.org/). The raw data supporting the conclusions of this article will be made available by the authors, without undue reservation.
