# Supplementary figures and images for: Integrative analysis of efferocytosis- and invasion-related genes as potential biomarkers and therapeutic targets in breast cancer
Source: Discov Oncol. 2025 Aug 5;16:1474. doi: 10.1007/s12672-025-03346-w (PMC12325836; doi:10.1007/s12672-025-03346-w)

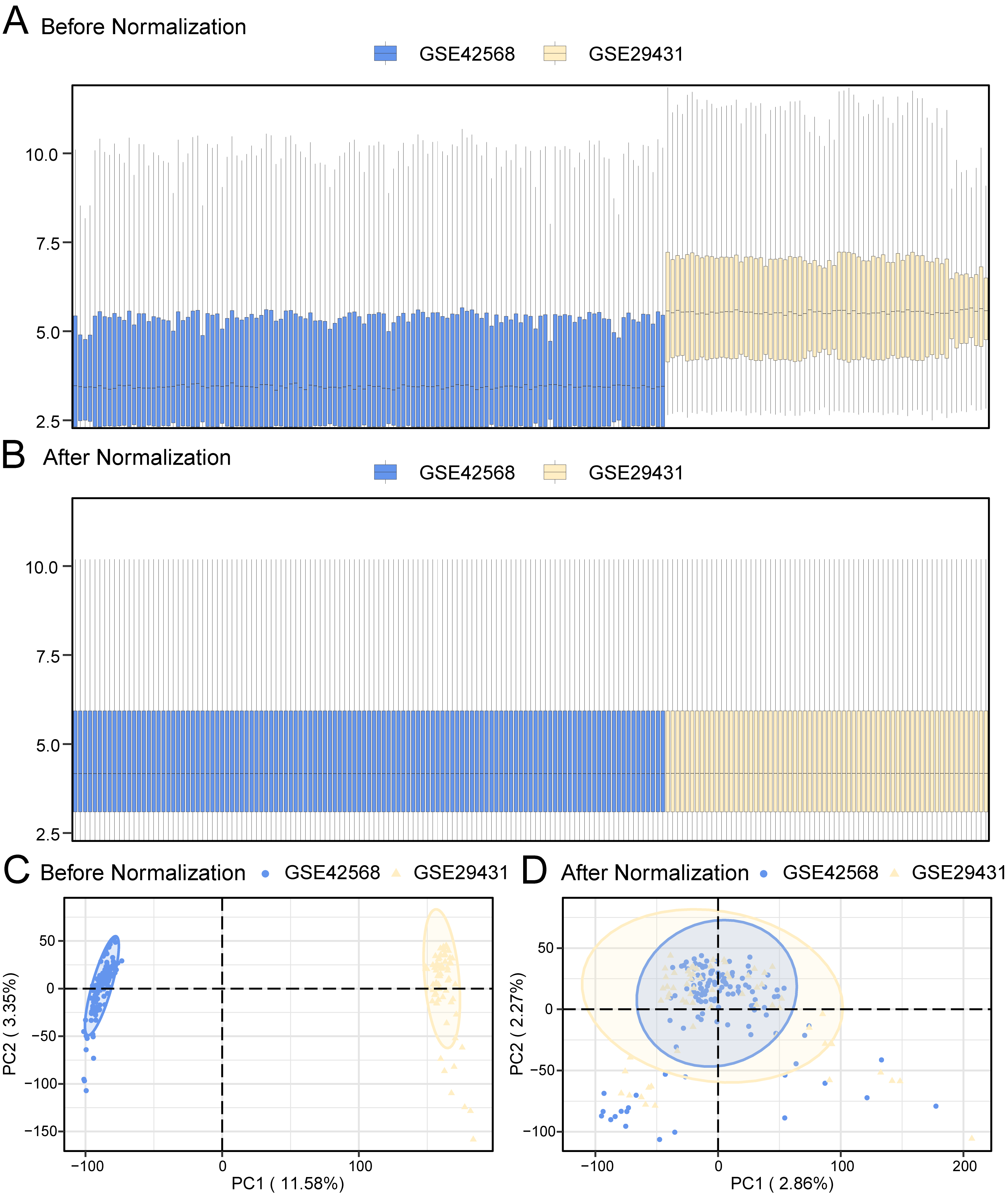

Supplement: Supplementary file 5 — Supplementary Material 5 [file 12672_2025_3346_MOESM5_ESM.tif]
